# An ethological analysis of close-contact inter-cat interactions determining if cats are playing, fighting, or something in between

**DOI:** 10.1038/s41598-022-26121-1

**Published:** 2023-01-26

**Authors:** N. Gajdoš-Kmecová, B. Peťková, J. Kottferová, V. Halls, C. Haddon, L. Santos de Assis, D. S. Mills

**Affiliations:** 1 Applied Research Centre, University of Veterinary Medicine and Pharmacy in Košice, Košice, Slovakia; 2 Workplace of Applied Ethology and Professional Ethics, University of Veterinary Medicine and Pharmacy in Košice, Košice, Slovakia; 3 International Cat Care, Tisbury, Wiltshire UK; 4 Gloucestershire, UK; 5 Animal Behaviour, Cognition and Welfare Group, Dept. of Life Sciences, University of Lincoln, Lincoln, UK

**Keywords:** Animal behaviour, Behavioural methods

## Abstract

Intraspecific social interactions in domestic cats are often categorised as affiliative or agonistic. However, public or professional assessment of encounters can have difficulty distinguishing rough-and-tumble play from true agonism. One possible issue is the potential occurrence of elements of both, play and agonism, within inter-cat play, for example when one cat wants to terminate a bout of play but the other seeks to continue the interaction, which subsequently may provoke more overt agonistic behaviour. To test this hypothesis, we conducted behavioural observations of 105 unique dyadic interactions of domestic cats (N = 210) captured on videos collected from owners and YouTube. We assessed cats for the frequency and duration of six behavioural elements. The dataset was reduced using PCA with a varimax rotation and factor scores were used to classify the population using hierarchical cluster analysis. To validate the identified clusters, the average scores of the constituent factors were compared and the data on interactions were labelled by four cat behaviour experts as “playful”, “intermediate” or “agonistic”. In addition, to evaluate properties of expert-labelled categories we used linear discriminant analysis followed by an ordinal regression. The results showed considerable convergent validity in factor distributions between clusters and expert-labelled groups: reciprocal wrestling was most closely associated with a group of playfully interacting cats, while vocalisation and chasing were associated with the agonistic group. The intermediate group, while having characteristics of both, was more closely related to the playful group than the agonistic group, with prolonged exchanges of interactive behaviours being a predominant feature. Thus, our findings support the suggestion of there being an intermediate category between mutual social play and agonism. This might escalate into a fully agonistic encounter, but does not necessarily reflect a break down in their social relationship but rather a short-term disagreement in social priorities.

## Introduction

Successful interpretation of social interactions between cats is an important part of meeting standards for the wellbeing of co-habiting cats in multi-cat households^1^ and helps to reduce the risk of additional undesirable behaviours such as house soiling problems. In this regard, to date, research has focused on issues such as clarifying the meaning of vocal or olfactory communication^2^, tail signalling^3,4^ but also understanding the principles of organisation of free-living cat colonies^5^. However, as recently reviewed, there are still many gaps in our knowledge of feline sociality, including a lack of cross-study consensus^6^. Perhaps one of the most overlooked issues that often concerns owners is distinguishing rough and tumble social play from agonistic interaction in cats. Some owners appear to consider inter-cat conflict as a normal or inevitable feature of the multicat household^7^, and conflict behaviours may be misperceived by the owners as a form of inter-cat play and thus not reported to problem behaviour professionals^7,8^. There appears to be a blurred line between playful and agonistic aggression between cats, as evidenced by (a) play-related aggressive behaviour being one of the cited contexts in the clinical veterinary behaviour literature for problems in cats^9,10^, and (b) inclusion of motor patterns from strictly functional behavioural expressions (including agonistic behaviours) as one of the general characteristics of play^11^. This problem is exacerbated by the great diversity in the terminology, definitions and points of reference used in relation to the description of play in cats and its classification—see Gajdoš-Kmecová et al.^12^ for a recent review of these issues. To address these concerns these authors proposed a psychobiological approach which includes the systematic inclusion of inferences about motivation and affect alongside a description of context^13^. This approach highlights distinctions relating to different forms of inter-cat play provide by their emotional basis. Thus, play involving one cat treating another as if it was a prey or object, is distinguished emotionally (SEEKING sensu Panksepp, 1998^14^) from mutual rough and tumble social play (hereafter “mutual social play”) which involves reciprocal activity and is pleasurable for both (PLAY sensu Panksepp, 1998^14^). However, it remains unknown how difficult it might be to apply this theoretical framework in real-world situations. Therefore, the aim of this study was to conduct an ethological analysis of various close-contact inter-cat interactions in order to identify categories of behaviour which might be helpful for distinguishing between agonistic and playful interactions and evaluate it for convergent validity with clinical behaviour assessments of the nature of the interactions. This information may not only provide further evidence for the value of a psychobiological diagnostic process^15^ but also provide a solid foundation for future recommendations concerning the management of inter-cat behaviour within the household.

## Methods

### Data collection and preparation

Videos of inter-cat interactions in natural domestic (indoor and outdoor) inhabited environments were obtained in two ways—retrospectively via “YouTube” and prospectively from cat owners.

The website (youtube.com) was browsed using specific search terms: “cats play fighting”, “cats play with each other”, “cats playing together” and “cats fighting”. Videos of clearly agonistic interactions between cats were also included (those where uninhibited biting, scratching and kicking, cat fur covered with blood and claws stuck in the other cat's skin were observed). Due to a pilot character of this study and preventing exclusion of what might be important, no selection criteria were used for the duration of the video included within the sample. No additional information about cats was collected nor assumed (e.g. sex of the cat based on the visual of the external reproductive organs or socialisation status based on cat's proximity to the observer/person taking the video). Videos were used in accordance with their Creative Commons policy. This method has been used previously by other researchers, e.g.^16^.

In response to an advert shared on social media (Facebook) cat owners who were interested in participating in the study contacted one of the authors via email. They were provided with a brief outline of what this study was about. If they wished to continue, they provided informed consent with a detailed explanation of their involvement in the study, signed by them. They could then provide videos of cats. Owners did not provide any personally identifying information other than that given in the informed consent and no information about cats in the videos was collected. Owners were requested not to provoke their cats in order to gain footage.

Videos from the web and from owners were excluded according to the following criteria: those which did not include inter-cat interaction (e.g. cat was only manipulating an object, cat was grooming itself etc.), those which included interaction of three and more cats, and videos which were of poor visual quality or had their original sound removed of replaced by other sound (e.g. music). To prevent pseudoreplication, videos of the same dyad of cats interacting were not included in the analysis. Videos from the web which were cut into short clips but were capturing the same cats within same interaction in the same environment were analysed as one video of the same dyadic interaction.

Each dyadic interaction was assigned a unique code to identify it within the analysis. Notes about age category of a cat were written down, with only “kitten” and “adult” distinguished. Kitten was defined as a clearly skeletally immature individual.

### Ethogram and behavioural observations

Based on preliminary observations of 30% of randomly selected videos from the sample, an ethogram of six high level behavioural categories was created (Table 1). This was initially based on ethograms used by other authors^17–19^ but some behaviours were added to provide a more detailed description of these categories. In order to increase reliability we focused on overt behaviours that could be easily viewed consistently, rather than subtle signals which might be obscured. Accordingly, only whole body behaviours were recorded within each of the six behavioural categories, without a specific focus on recording the ear and tail movements. These body parts could be dynamic or static during recording (see Table 1).

**Table 1:** Ethogram of inter-cat interactions.

Inactive body posture	Head and torso of the cat are motionless and in a specific position (e.g. crouch, lying, siting, standing, rear)
Wrestling	Cat engages in physical contact with another cat, whereby the focal cat appears to struggle with the other cat. This can include pulling the cat toward itself with its forelegs and perform raking movements with the hind legs. (reversed wrestling, reversed half-wrestling, parallel wrestling, parallel half-wrestling, within wrestling: foreleg movements, bites, snap bites, non-injurious biting, rake, kick)
Chasing	Cat runs rapidly in pursuit of other cat or cat runs away from other cat (flee) or one cat travels closely behind a cat (follow)
Other interactive activities	Activity of cat directed towards another cat. (allogroom, approach, arch back, avoid, belly-up, displace, face-off, foreleg movements directed towards other cat, horizontal leap, lordosis, mount/clasping, neck flex, piloerection, pounce, retreat, side step, sniffing other cat, stand up, stalk, vertical stance, roll on back)
Non-interactive activities	Activity of cat directed towards itself or inanimate object. (manipulate object, drink, head shaking, jumping, self-licking, running, trotting, walking)
Vocalisation	Cat produces sounds or calls, originating from the throat and mouth (growl, hiss, snarl, spit, yowl, mew, gurgle)

Frequency and duration of behaviours from the six behavioural categories defined in the ethogram were recorded using Solomon coder. Behaviour of each cat from the dyad was recorded using focal animal sampling method and 0.2 s recording time intervals.

An intra-observer reliability test was conducted to confirm consistency of behavioural coding—10% of videos were coded a second time by the same observer 2 months later and Spearman correlation coefficient was calculated for the duration and frequency of each of the six behavioural categories in the two sets of data. Spearman correlation coefficients ≥ 0.7 were determined as sufficient to continue with further analysis.

Duration (in seconds) and frequency of each behavioural category for each cat in the dyad (i.e., 12 variables per cat) were standardised by converting them into proportions (dividing rate of the behaviour, either frequency or duration, by duration of observable time in seconds).

In addition, four authors—two based in academia (DM, NGK) and two from the field of animal behaviour consulting (CH, VH)—labelled the dyads of interacting cats as either “playful”, “agonistic”, “intermediate” (mixed interaction during which behaviour of at least one cat had characteristics of both) and “not sure”. Three categories provided sufficient information on the possible emotional state of each of the cats—positive for playful, negative for agonistic and mixed for the intermediate category. Each dyad was then labelled on the basis of the majority agreement (3 out of four or two out of three in case that one labelled it as “not sure”). Interactions where there was an even split of opinions (N = 9) were reassessed in a second round, by the same four experts with a note describing the two categories over which there had been disagreement reported previously. If this did not resolve the issue, the case was removed from the sample (N = 4, split between playful and intermediate category, N = 3 for agonistic versus intermediate categories, N = 1).

The dataset consisting of standardised score values for the 12 variables for each cat of the dyad, supplemented by the labels assigned to each interaction according to the expert opinion, was then subjected to statistical analysis.

### Statistical analysis

The dataset was reduced using principal components analysis using a varimax rotation. Inflection on the screen plot graph was used to determine the number of components to extract (Supplementary Fig. 1).

Subjects were then assigned factor scores based on the weighing of the constituent measures making up each component [e.g., Factor 1 score = (F1 loading value for first variable*z-score value for first variable) ± (F1 loading value for second variable* z-score value for second variable) ± (etc. per each of 12 variables)].

Using the dataset of factor scores assigned to each subject (cat), hierarchical cluster analysis with Ward’s linkage was then used to describe the relationships between individuals within the population.

Mean factor scores and standard deviations were then calculated for each cluster identified. These values were then used to characterise the behaviour of subjects in clusters.

Pearson's chi-square tests were used to determine the significance of potential correlates which could help to suggest the type of interaction: presence of kittens, expert consensus, and presence of complete dyads within the same cluster.

To validate and further understand the clusters identified by the clustering algorithm and our interpretation thereof, the distributions of the six components were compared between the clusters and the data associated with the expert labels “playful”, “intermediate” and “agonistic”. Boxplots and a confusion matrix were used to this end. It was not considered appropriate to generate complex metrics beyond the confusion matrix, as we do not expect the clustering to be as precise as or replace the skilled professional, but rather to guide us towards broad concepts. A preliminary linear discriminant analysis to establish unidimensionality, followed by an ordinal regression were then used to understand the properties of our expert-labelled groups, including inferential statistics to help quantify uncertainty.

Statistical analyses were conducted using R statistical software and a “psych” package.

### Ethics statement

The study was reviewed and approved by College of Science Research Ethics Committee, University of Lincoln (CoSREC431). Written informed consent was obtained from the owners for the participation of their animals in the study. All methods were performed in accordance with the relevant guidelines and regulations of the University of Lincoln and University of Veterinary Medicine and Pharmacy in Košice.

## Results

### Sample characteristics

One hundred and sixty-five videos were collected, with 63 videos from owners and 102 videos downloaded from YouTube. These videos captured more than 250 different events with cats. One hundred and fourteen videos of unique dyadic inter-cat interactions (228 cats) fulfilled the inclusion criteria for further behavioural analysis. Two others were removed due to missing sound in the video.

After expert assessment, 4 dyads (8 cats) were removed from the sample due to split opinions. This split in opinions was between either the playful and intermediate categories (N = 3 dyads) or agonistic and intermediate categories (N = 1 dyad), but not between the playful and agonistic categories. Three interactions (6 cats) were removed since at least two assessors labelled them as “not sure” (comments were made that they showed sexual behaviour or only a grooming session between cats). Thus, a final sample of 105 interactions with 210 cats was subjected to statistical analysis.

Thirty-eight cats (18.1%) from the final sample (N = 210) were clearly skeletally immature individuals (kittens). Fourty-one (39%) interactions were situated in outdoor environment and 64 (61%) took place in indoor environment conditions. Average video duration was 2 min and 2 s (0:11–11:08, Median 1:22). More than a half of the cats in our sample (N = 118, 56.2%) were described by experts as playful in their interaction, 15.2% (N = 32) were labelled as intermediate and 28.6% (N = 60) cats were assigned with “agonistic” label.

### Principal component analysis and cluster analysis

Six principal components were retained, with each factor explaining a similar amount (between 11 and 18%) of the variance (Table 2). Within factors, variables with loadings greater than 0.7 were selected (Table 2). Factors were named according to variables loading most heavily on them as follows: F1 Wrestling vs. inactivity, F2 Vocalising, F3 Chasing, F4 Non-interacting, F5 Recurring interactivity, F6 Prolonged interactivity.

**Table 2:** Results for six retained factors, bold items suggest selected variables (loadings ≥ 0.7).

	F1	F2	F3	F4	F5	F6
FIBP	− 0.28	− 0.01	0.19	0.12	**0.84**	− 0.08
FW	**0.83**	− 0.08	− 0.07	− 0.10	0.36	− 0.01
FCH	0.02	0.00	**0.94**	0.00	0.21	0.04
FOIA	0.35	− 0.04	0.24	0.05	**0.82**	0.19
FNIA	− 0.03	− 0.10	0.03	**0.88**	0.26	− 0.06
FV	− 0.14	**0.96**	− 0.02	− 0.12	− 0.01	− 0.02
DIBP	− **0.72**	0.33	− 0.08	− 0.19	0.26	− 0.44
DW	**0.82**	− 0.25	0.01	− 0.20	− 0.27	− 0.27
DCH	− 0.04	− 0.05	**0.95**	− 0.04	0.11	− 0.01
DOIA	− 0.05	− 0.01	0.02	− 0.09	0.07	**0.99**
DNIA	− 0.09	− 0.14	− 0.06	**0.93**	− 0.10	− 0.02
DV	− 0.19	**0.95**	− 0.04	− 0.12	− 0.04	− 0.01
Names assigned to factors	Wrestling vs. inactivity	Vocalising	Chasing	Non-interacting	Recurring interactivity	Prolonged interactivity
Proportion of variance explained	0.18	0.17	0.16	0.15	0.15	0.11

FIBP, Frequency of Inactive body posture; FW, Frequency of Wrestling; FCH, Frequency of Chasing; FOIA, Frequency of Other interactive activities; FNIA, Frequency of Non-interactive activities; FV, Frequency of Vocalisation; DIBP, Duration of Inactive body posture; DW, Duration of Wrestling; DCH, Duration of Chasing; DOIA, Duration of Other interactive activities; DNIA, Duration of Non-interactive activities; DV, Duration of Vocalisation.

**Figure 1:**
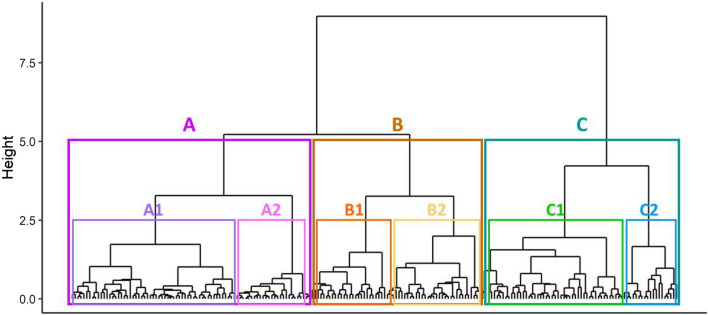
Relationship between the individuals (bottom line) characterised by their factor scores described by a dendrogram of hierarchical cluster analysis with Ward’s linkage.

Cluster analysis revealed that the population could be broadly divided into three main populations (A, B and C), with six subgroups (A1, A2, B1, B2, C1 and C2) at a lower level (cut at 2.5) (Fig. 1). For the mean loadings and standard deviations (SD) of factor scores for each the three main clusters see Table 3 [Colour scale coding aids navigation from positive (green) to negative (red) values for means. For the mean loadings and standard deviations of factor scores for the six subclusters see Supplementary Table 1.

**Table 3:**
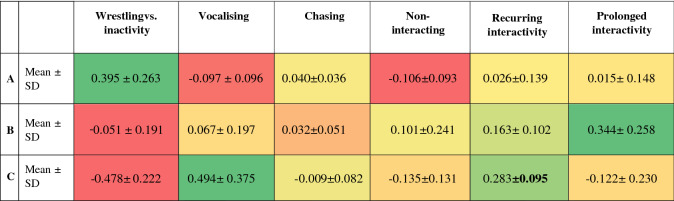
Mean factor scores of the clusters evaluated at the higher level. Low standard deviation suggesting importance of the mean score is highlighted in bold.

### Cluster characteristics

#### Cluster A

This cluster included 39.5% (83) of cats from our sample. It was characterised by the highest score for Wrestling vs. Inactivity (PC1, 0.395); a negative score for Non-interacting (PC4, − 0.106); and close to zero scores for Vocalising (PC2, − 0.097), Chasing (PC3, 0.040), Recurring interactivity (PC5, 0.026), and Prolonged interactivity (PC6 0.015), (Table 3). This suggests that this is a cluster of wrestling and non-vocalising cats. Kittens (30.21% of cats in the group, SR = 3.66***, χ^2^ = 18.647), cats from within the same dyad (86.7%, SR = 4.18***, χ^2^ = 19.126) and cats from dyads assessed as a playful interaction (83.1%, SR = 6.36***, χ^2^ = 74.561) were significantly overrepresented in this cluster, with cats considered to be involved in agonistic dyads (8.4%, SR = − 5.22***, χ^2^ = 74.561) strongly underrepresented here (for details see Table 4).

**Table 4:** Pearson chi-square tests for categorical variables of higher level of hierarchical analysis’s clusters 2 d.f., *p ≤ 0.05, **p ≤ 0.01, ***p ≤ 0.001.

	A	B	C	Results (χ^2^, p value)
**Kittens** **vs. Adults**				
**Kittens**				χ^2^ = 18.647 p ≤ 0.001
Observed (% of group)	25 (30.1%)	11 (18.6%)	2 (2.9%)	
Expected	15.0	10.7	12.3	
Standardised residual	3.66***	0.13	− 3.95***	
Standardised residual—p value	**0.0003**	0.8972	**p ≤ 0.001**	
**Adults**				
Observed	58 (69.9%)	48 (81.4%)	66 (97.1%)	
Expected	68.0	48.3	55.7	
Standardised residual	− 3.66***	− 0.13	3.95***	
Standardised residual—p value	**0.0003**	0.8972	**p ≤ 0.001**	
**Cats in original dyads vs. Not in dyad**				
**In dyad**				χ^2^ = 19.126 p ≤ 0.001
Observed	72 (86.7%)	32 (54.2%)	44 (64.7%)	
Expected	58.5	41.6	47.9	
Standardised residual	4.18***	− 3.22**	− 1.27	
Standardised residual—p value	**p ≤ 0.001**	**0.0013**	0.2046	
**Not in dyad**				
Observed	11 (13.3%)	27 (45.8%)	24 (35.3%)	
Expected	24.5	17.4	20.1	
Standardised residual	− 4.18***	3.22**	1.27	
Standardised residual—p value	**p ≤ 0.001**	**0.0013**	0.2046	
**Playful vs. Intermediate vs. Agonistic interaction**				
**Playful**				χ^2^ = 74.561 p ≤ 0.001
Observed	69 (83.1%)	34 (57.6%)	15 (22.1%)	
Expected	46.6	33.2	38.2	
Standardised residual	6.36***	0.26	− 6.90***	
Standardised residual—p value	**p ≤ 0.001**	0.7931	**p ≤ 0.001**	
**Intermediate**				
Observed	7 (8.4%)	15 (25.4%)	10 (14.7%)	
Expected	12.6	9.0	10.4	
Standardised residual	− 2.22*	2.57*	− 0.15	
Standardised residual—p value	**0.0266**	**0.0103**	0.8819	
**Agonistic**				
Observed	7 (8.4%)	10 (16.9%)	43 (63.2%)	
Expected	23.7	16.9	19.4	
Standardised residual	− 5.22***	− 2.33*	7.69***	
Standardised residual—p value	**p ≤ 0.001**	**0.0198**	**p ≤ 0.001**	
Total (N)	83	59	68	

Significant values are in bold.

The two subclusters of Cluster A were distinguished by the amount of wrestling, vocalisation and interactivity. Cats in Subcluster A1 were wrestling less (PC1, 0.258, p ≤ 0.001) vocalising more (PC2, − 0.052, p ≤ 0.001) and interacting more (PC5, 0,097 and PC6, 0.073) than cats from Subcluster A2 (PC1, 0.670 and PC2 − 0.194, PC5, − 0.128, PC6, − 0.111, p ≤ 0.001). Cats from dyads described as a playful interaction (86% of cats in the group, SR = 5.31***, χ^2^ = 101.02) and from within the same dyad (77.2%, SR = 3.42***, χ^2^ = 27.165) were overrepresented in A1, while cats described as from an agonistic dyad were highly underrepresented here (5.3%, SR = − 4.56***, χ^2^ = 101.02). Although in A2, kittens (38.5%, SR = 2.88**, χ^2^ = 28.578) and cats from within the same dyad (84.6%, SR = 2.93**, χ^2^ = 27.165) were overrepresented, this was with lower significance value (p ≤ 0.01, for detailed results see Supplementary Tables 1–4).

#### Cluster B

Cluster B comprised of 28.1% (59) cats, and was more closely associated with Cluster A than C. When compared with the other two clusters it had the highest score for Prolonged interactivity (PC6, 0.344, p ≤ 0.001) and was also characterised by relatively high scores for Recurring interactivity (PC5, 0.163) and generally low scores for Wrestling vs. inactivity (PC1, − 0.051), Vocalising (0.067), Chasing (0.032) and Non-interacting (0.101, Table 3). Cats within this cluster were therefore interacting for relatively prolonged periods while interaction was often characterised by pauses within the interaction. Inclusion of both cats from within their original dyad was underrepresented in this cluster (54.2% of cats in the group, SR =—3.22**, χ^2^ = 19.126), while dyads classified as being from an intermediate type of dyadic interaction were overrepresented here (25.4%, SR = 2.56*, χ^2^ = 74.561). Cats from agonistic dyads were underrepresented in Cluster B (16.9%, SR = − 2.33*, χ^2^ = 74.561, p ≤ 0.05, for details see Table 4).

Cluster B was divided into two subgroups which could be distinguished based on the type and duration of pauses in cats' interactions. Within the B1 subcluster interactions were characterised by the highest mean score for Non-Interacting (PC4, 0.344, p ≤ 0.001) with an underrepresentation of cats from agonistic dyads (3.8% of cats in the group, SR = − 2.98**, χ^2^ = 101.02). The B2 subcluster had the highest mean score for Prolonged interactivity (PC6, 0.513, p ≤ 0.001) with cats from the intermediate type of dyadic interaction overrepresented (30.3%, SR = 2.62**, χ^2^ = 101.02, for details see Supplementary Tables 1–4).

#### Cluster C

Cluster C included 32.4% (68) of cats from our sample It was characterised by the highest scores among all clusters for Vocalising (PC2, 0.494, p ≤ 0.001) and Recurring interactivity (PC5, 0.283, p ≤ 0.001), but also by the lowest score for Wrestling vs. inactivity, suggesting Prolonged inactivity (PC1, − 0.478, p ≤ 0.001). Therefore, these cats were vocalising during a recurring interaction and were rather inactive for longer periods, during their interaction. Cats from the agonistic dyadic interaction were overrepresented (63.2% of cats in the group, SR = 7.69***, χ^2^ = 74.561), while kittens (2.9%, SR = − 3.95***, χ^2^ = 18.647) and cats from playful interaction dyads (22.1%, SR = − 6.9***, χ^2^ = 74.561) were markedly underrepresented in this cluster.

Cats from the C1 subcluster were less often inactive for longer periods (PC1, − 0.402, p ≤ 0.001) and were less vocalising (PC2, 0.287, p ≤ 0.001) during their interaction than cats from the C2 subcluster, where mean scores for Wrestling vs. inactivity, Vocalisation were at their extreme (PC1, − 0.674, PC2, 1.026, p ≤ 0.001). Cats from agonistic dyads were markedly overrepresented in both subclusters (C1:49%, SR = 3.61***, C2: 100%. SR = 7.23***, χ^2^ = 101.02), while cats from playful interaction dyads were markedly underrepresented in them (C1: 30.6%, SR = − 4.12***, C2: 0, SR = − 5.18***, χ^2^ = 101.02). Kittens were also underrepresented in the C subclusters (C1: 4.1%, SR = − 2.91**, C2: 0, SR = − 2.15*, χ^2^ = 28.578, for detailed results see Supplementary Tables 1–4).

### Validating the clusters

The confusion matrix in Table 5 shows a degree of agreement between the expert-labels (“playful”, “agonistic” and “intermediate”) and the clusters, with 72% of agonistic cases being found in Cluster C, 58% of playful cases in Cluster A and 47% of intermediate cases in Cluster B.

**Table 5:**
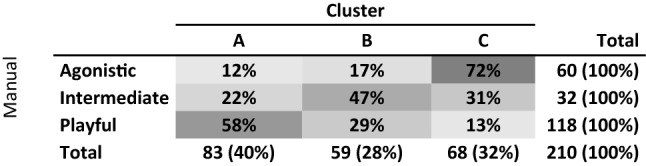
Confusion matrix comparing manual expert-labels with those assigned by clustering algorithm.

**Figure 2:**
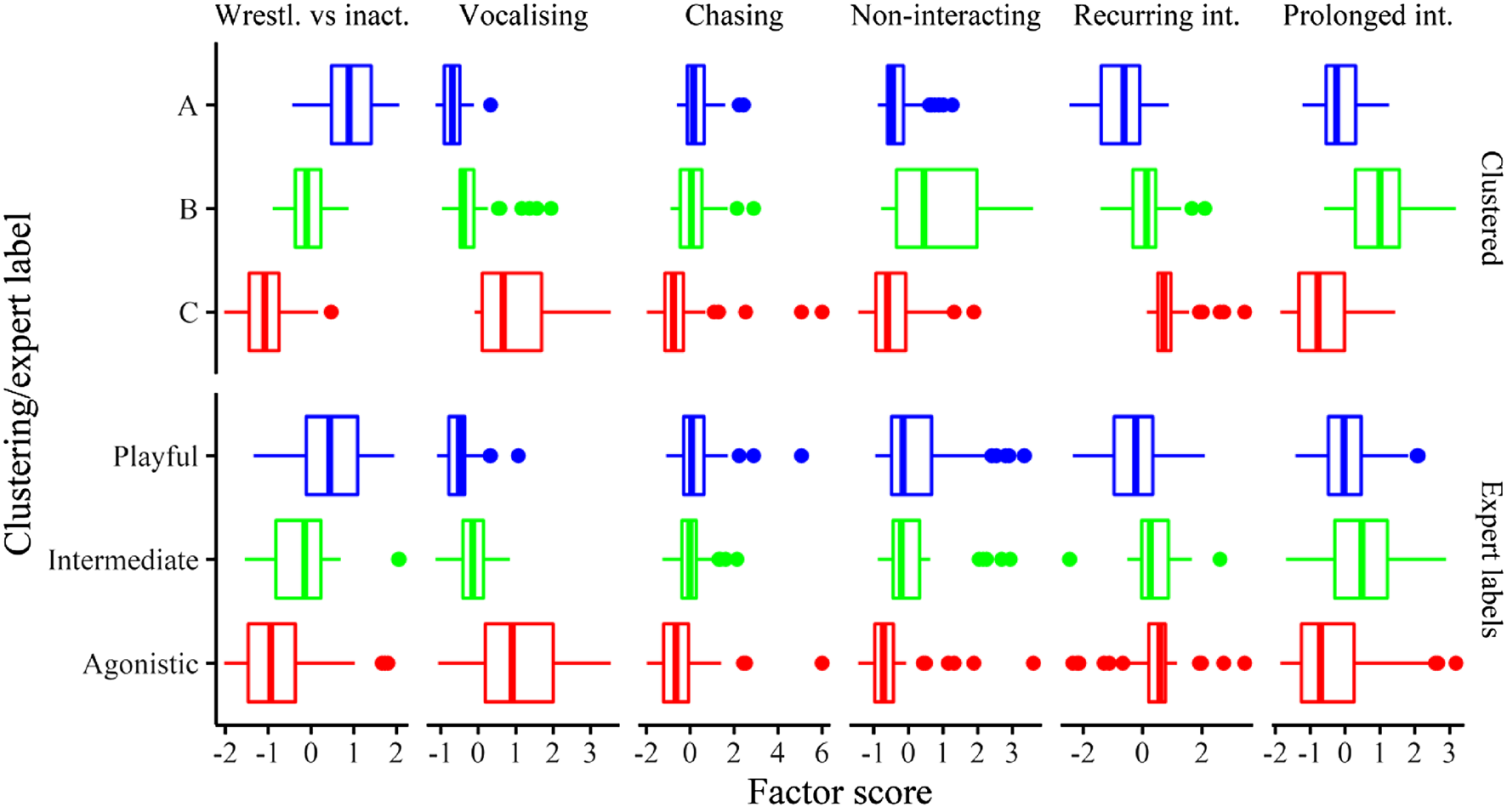
Boxplot comparing marginal distributions of clustered vs expert-labelled groups.

A clustering algorithm based on six variables is unlikely to capture all of the subtleties of a human watching an entire video, and so the descriptive summary results including distributions were visually compared between the two approaches. The boxplot in Fig. 2 illustrates the marginal distribution of each of the factors between the clusters and the expert labels. Each of the boxplots in the top three rows captures the marginal distributions of the respective factor, across the three clusters, while those in the bottom three rows captures the distribution for the three expert-labelled groups. Boxes of the same colour represent the cluster/expert labels that we suggest align with each other (for example, cluster A and the “Playful” category are both coloured in blue). Similarity in the aligned groups indicates matching between the mathematical clusters and expert-labelled groups. There appears to be a remarkable level of agreement for all factors except the “Non-interacting” factor. We might expect the expert-labelled groups to be more dispersed, since the clusters are based on drawing strict boundaries in this same data, and the expert labels are not. While this doesn’t explain which combinations of variables are considered important, it provides convergent validity for our interpretation of the clusters.

### Further analysis of expert categories

A linear discriminant analysis of the six factors, across the three expert-labelled groups, indicated that most of the discriminatory power between the groups lay along one single linear discriminant (the first linear discriminant accounted for 92% of the trace). The boxplots in Fig. 2 also support the idea that the “intermediate” category lies part-way between the “agonistic” and “playful” categories (with the possible exception of “Prolonged interactivity”). It was therefore considered useful to obtain some inferential statistics via an ordinal regression with “intermediate” coded as the middle category. Results of this regression can be found in Table 6. All factors were standardised, so that coefficients can be compared; negative coefficients represent more agonistic behaviours, while positive coefficients relate to playful behaviours. Of the agonistic behaviours, vocalisation and chasing are statistically significant. Recurring interactivity, on the other hand was “significantly playful”. By far the clearest of these is the role of vocalisation in agonistic behaviour.

**Table 6:** Ordinal regression of expert labels onto factor scores.

Predictors	Coefficient	Odds Ratios	CI	p
A|I	− 0.92			
I|P	0.41			
Wrestling vs. inactivity	0.84	2.32	0.82–6.52	0.110
Vocalising	− 3.49	0.03	0.01–0.12	** < 0.001**
Chasing	− 0.53	0.59	0.36–0.98	**0.040**
Non-interacting	0.20	1.22	0.79–1.95	0.391
Recurring Interactivity	1.35	3.84	1.67–9.47	**0.003**
Prolonged Interactivity	− 0.15	0.86	0.59–1.25	0.416
R^2^ Nagelkerke	0.584			

Significant values are in bold.

Individual logistic regressions showed similar slopes for individual cut-off values of the ordinal regression, so that the assumption of homogeneity of slopes seems to be satisfied.

## Discussion

The results of this study suggest distinction of three types of close animated inter-cat interactions: playful, agonistic and an intermediate form involving elements of playful wrestling, but also the vocalisation and chasing typical for agonistic interactions. This is in line with our previously proposed theoretical framework suggesting that play between cats may be mutual, but it may also be unbalanced, for example predatory play where one cat is the “prey”, or if one cat does not wish to play at a given time, in which case it may respond to the playful intent of the other with an agonistic response. The latter may occur before play has begun, as one cat’s solicitation is aggressively declined, or during a playful bout when one cat decides to end the interaction^12^. Thus, the common binary classification of inter-cat interactions as either affiliative or agonistic^20–24^ might be misleading.

Of the three types of interaction described, the most distinctive is the agonistic one described by the experts, with many of these cats in Cluster C. This type of interaction was characterised by vocalisation, chasing, recurring interactivity and prolonged inactivity. The occurrence of vocalisation was particularly important in this regard, being highest in this cluster and also significant in the ordinal regression analysis. It is well-known that feline intraspecific vocalisation is a common feature of agonistic encounters, but it also occurs as part of sexual and mother-young communication^2^, which were not considered in this study. Thus, we confirmed anecdotal suggestions that extensive vocalisation can help to distinguish agonistic from playful interactions^7,25^. Chasing was also an important behaviour in our study, explaining the third greatest proportion of variability in our data, and significantly associated with the agonistic dyads described by the experts. This is consistent with previous observations of chasing as part of agonistic behaviour repertoires^23,24,26^. However, it should be noted that chasing (especially if extensively reciprocated) can also be a part of mutual social play^17,27,28^. In context of our results, its increase during an interaction might indicate a shift in motivation (of at least one of the previously mutually playing cats) towards a desire to stop the interaction. Running away being potentially used to terminate the interaction, as proposed by Gajdoš Kmecová et al. in their model of cat play. The agonistic nature of the interactions of cats in Cluster C is further supported by the pattern of recurring interactivity and prolonged inactivity (Wrestling vs.inactivity factor). It has previously been noted that beside ritualised vocal duels, agonistic interactions mostly consist of threatening postures^24,26^, and offensive/defensive behaviours [e.g. piloerection, cuffing, or ear and tail movements^20,23^], some of which were included within our category of other interactive behaviours, and thus were part of our Recurring interactivity factor. Moreover, submissive behaviours, represented by, for example crouched or half-sitting postures^24,26,29^, which are also a common feature of agonistic interactions, were recorded as a form of inactive behaviour which were an important factor in our analysis (Wrestling vs. inactivity factor). “Staring”, which occurs during interactive behaviours, while in still position is another commonly reported behaviour used within inter-cat conflict^20,23,29^. Together with ear and tail movements, behaviours not recorded in this study, they can be difficult for owners to observe, but may be important to explaining the emotional state of interacting cats (See: Bennet et al.^30^). Therefore, to confirm the value of the recurring interactivity and prolonged inactivity patterning, staring and movements of ears and tail should be recorded in the further studies alongside other body movements and a more refined classification of vocalisation.

The other widely recognised type of exchange, that has been described previously was the mutual playful one represented by Cluster A, which had an over-representation of the playful dyads identified by the experts. Frequent and long-lasting wrestling was a particular feature characterising cats in this cluster and the playfully interacting dyads identified by the experts. Cats avoid close physical contact during truly agonistic interaction, using defensive and offensive behaviours that involve little direct contact^24,26,29^. Thus the cats in this cluster with their prolonged wrestling were unlikely to be acting agonistically towards each other. However, wrestling (despite its high coefficient value) was not a significant characteristic of playfully interacting dyads, and given its potentially agonistic associations, wrestling should not be used by itself as an indicator of playfulness. Reciprocity within mutal social play wrestling often takes the form of role reversal with regards to who is on the top, and may occur both between numerous bouts and/or within longer bouts of play^11^. It also worth noting that kittens were significantly overrepresenated in this cluster, with the majority of reciprocally wrestling kittens being within A2, suggesting this might represent the kitten form of play. It has previously been noted that wrestling significantly increases from 4–7 weeks of age to the 8–12 weeks of age period^31^. There is a notable lack of literature on adult cat play, so it is worth noting that the behaviour in A2 was distinguished from A1 not only by more wrestling but also less vocalisation, suggesting adult play may differ from kitten play in other behavioural features.

Cluster B is described as an intermediate type of action, and corresponds most closely to the intermediate dyads described by the experts, and was characterised by features associated with both playful and agonsitic interactions. The most distinctive characteristic of this group was prolonged interactivity. The interactive behaviour category included behaviours previously described as part of mutual social play in kittens [such as belly-up, side-step, face-off, or pounce^17^], but also reproductive-like behaviours [e.g. mount, lordosis^18^], agonistic and aggressive behaviours [e.g. arch back, avoid, retreat, piloerection^18,32^] and behaviours belonging to multiple functional categories [e.g. allogrooming^33^, bite^27^, chase^23^]. From the analysis presented here it is not possible to say exactly which behaviours made up the interactions here, but from the experience of the observers it can be said it was not simply a seemingly playful exchange of belly-ups and face-offs. It featured interactive elements such as uni-directional pats followed by hiss and non-reciprocated chasing. i.e. acts commonly associated with enticement to play which were not reciprocated, or attempts to end a play bout. This is consistent with the finding that recurring interactivity was highest within the agonistic group. Morevoer, it is in line with the anecdotal report that play-fighting is characterised by plenty of gaps between bouts of interaction^34^. It is worth noting that there is no scientific evidence for metasignals associated with playing cats, which would help individuals end a playful interaction without hostility^12^. However, one possible explanation of this patterning could be that cats use inactive breaks within a playful interaction to reassess a partner's interest in continuing and thus, reduce the risk of escalation into serious agonism. As mentioned above, during these breaks, ears and tail movements may indicate what will follow and thus recording these movements in future studies as part of a more detailed ethogram would be useful to test this hypothesis. The non-interactivity factor was paricularly important especially in the definition of the B1 subcluster. This included behaviours of possibly different motivations and emotions. While some are emotionally positive [e.g. object play, locomotor play^14^], others are thought to be stress-related [e.g. lip-licking, head-shaking, auto-grooming^35^] and some might be considered relatively neutral (e.g. drinking, walking, running). Refinement of this non-interactive behavioural category and recording of more detailed categories or even behaviours might in the future provide valuable information which could help us to distinguish further subtypes of intermediate interactions.

It is worth noting that this is, to the authors’ knowledge, the first ethological analysis to specifically focus on inter-cat interactions in domestic settings. Due to its piloting character, no additional information about the cats (e.g. age, socialisation level, sex) were obtained. We acknowledge that further studies would benefit from collection of such data, however for some, such as socialisation level, this might not be the truth. Even if this information could be collected using questionnaire surveys, in many owned cats this information is missing, and tests of socialisation are notoriously unreliable. Based on current literature^36,37^ we can only suggest that most likely, in this and future studies, all cats captured on the videos by observing humans (either owners or people passing by the interacting cats) would be domestic cats with varying degree of contact with and acceptance of people, and on a lifestyle spectrum from pet/household cats to street cats, while feral cats would likely not be included within the sample, since these avoid people and are living in inhabited areas. This perhaps is the essence of what we mean by describing the work as being in a domestic setting. A simple ethogram of only six behavioural categories was used and these broad categories might be considered another limitation of this study. However, our use of relatively easily identifiable acts and data reduction method shows how relatively simple behaviours along with attention to their associations and patterning can provide potentially valuable insight into a common challenge relating to the evaluation of cat behaviour.

## Conclusion

Close inter-cat interactions should be considered under three groupings—playful (affiliative), intermediate and agonistic. When cats are young and when they are wrestling and not vocalising, they are most likely playing. On the other hand, when there are extended inactive breaks, vocalisation and chasing such interaction might not fulfil requirements for the mutual social play and is balanced by a degree of agonistic response. However, the amount of reciprocity in the exchanged behaviours should also be noted when evaluating the interactions between two cats by their owners or professionals during the diagnostic process. The more reciprocal the interaction is without agonism, then the closer the interaction is to true mutual social play^12^. This sort of structured objective approach to behaviour assessment, allows for the rational and scientific inference of the most probable emotional-motivational state of each of the interacting cats^38^. It is important to recognise that interactions may differ from day to day or even from one occasion within a day to the other, as proximate needs and wants vary. Thus, a single incident does not predict the relationship, i.e. even a few agonistic encounters between the two cats may not influence their general long-term relationship. For example, when two cats often rub against each other, allogroom, tend to sleep in close contact with one another, share resources and greet each other with ears erect^4,22,39^ occasional agonistic exchanges may not be an issue the owner should be too worried about. However, if the signs that cats are part of the same social group are ambiguous, e.g. when cats sleep near to each other but never in a close physical contact, allorubbing is not often present and allogrooming sessions often end up in agonism, and their inter-cat interactions are only occasionally reciprocally mutual, including little or no wrestling and some vocalisation, this might suggest a tension within their relationship with risks for physical and mental health^39^. Therefore, our findings (by focusing on general overt behaviours, rather than subtleties which might require some skill to detect in real time) provide valuable practical evidence which can be used to help owners detect signs of intercat tension in its early stages. Earlier detection and presentation to a clinical behaviour professional can be expected to be more likely to result in successful management of the relationship and prevent major issues which might lead to the relinquishment and/or euthanasia of one or both cats.

## Supplementary Information

Supplementary Information.

## Data Availability

The datasets used and/or analysed during the current study available from the corresponding author on reasonable request.
