# Supplementary material for: An ethological analysis of close-contact inter-cat interactions determining if cats are playing, fighting, or something in between
Source: Sci Rep. 2023 Jan 26;13:92. doi: 10.1038/s41598-022-26121-1 (PMC9879969; doi:10.1038/s41598-022-26121-1)
Supplement: Supplementary file 1 — Supplementary Information. [file 41598_2022_26121_MOESM1_ESM.docx]

**An ethological analysis of close-contact inter-cat interactions determining if cats are playing, fighting, or something in between**

Supplementary Information

Supplementary Figure 1 Parallel analysis scree plot


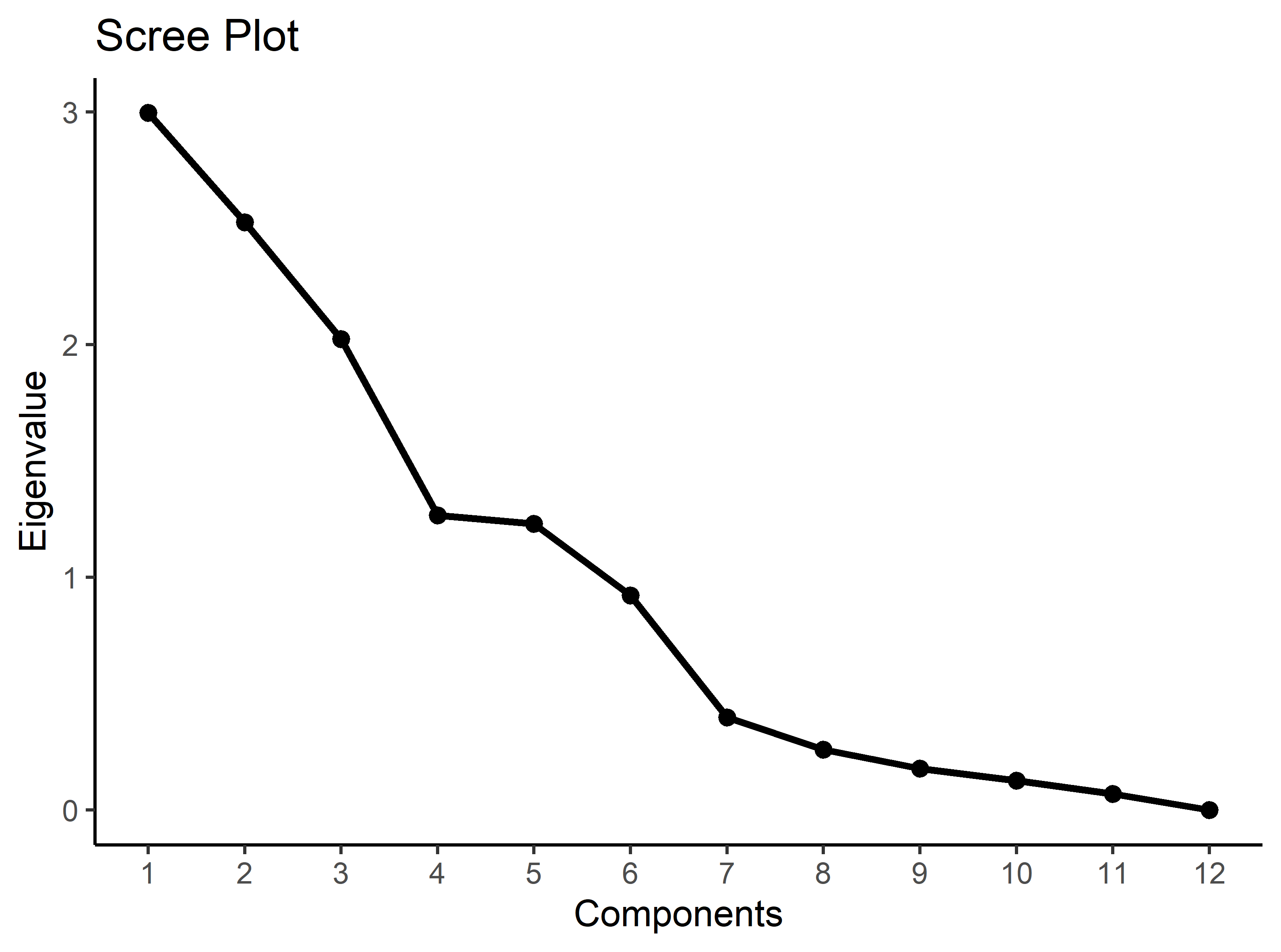


Supplementary Table 1 Mean factor scores of the clusters evaluated at the lower level (cut at 2,5). Low standard deviations suggesting importance of the mean score are highlighted in bold.

|  |  | Wrestling vs. inactivity | Vocalising | Chasing | Non-interacting | Recurring interactivity | Prolonged interactivity |
| --- | --- | --- | --- | --- | --- | --- | --- |
| A1 | Mean | 0.258 | -0.052 | 0.041 | -0.085 | 0.097 | 0.073 |
|  | SD | 0.188 | 0.081 | 0.039 | 0.090 | 0.099 | 0.125 |
| A2 | Mean | 0.697 | -0.194 | 0.037 | -0.152 | -0.128 | -0.111 |
|  | SD | **0.096** | **0.036** | 0.030 | 0.082 | 0.075 | 0.115 |
| B1 | Mean | -0.101 | -0.012 | 0.016 | 0.344 | 0.153 | 0.128 |
|  | SD | 0.208 | 0.091 | 0.057 | 0.134 | 0.124 | 0.154 |
| B2 | Mean | -0.012 | 0.129 | 0.045 | -0.090 | 0.171 | 0.513 |
|  | SD | 0.169 | 0.234 | 0.041 | 0.075 | 0.083 | **0.187** |
| C1 | Mean | -0.402 | 0.287 | 0.004 | -0.088 | 0.283 | -0.112 |
|  | SD | 0.191 | 0.152 | 0.085 | 0.123 | **0.087** | 0.223 |
| C2 | Mean | -0.674 | 1.026 | -0.041 | -0.255 | 0.284 | -0.149 |
|  | SD | 0.172 | 0.217 | 0.062 | 0.044 | 0.116 | 0.253 |

Supplementary Table 2 ANOVA and Tukey pairwise comparisons between the three clusters, separately for each factor.

|  |  | **Wrestling vs. inactivity** | **Vocalising** | **Chasing** | **Non-interacting** | **Recurring Interactivity** | **Prolonged Interactivity** |
| --- | --- | --- | --- | --- | --- | --- | --- |
|  |  |  |  |  |  |  |  |
| A | *M* | 0.395 | -0.097 | 0.040 | -0.106 | 0.026 | 0.015 |
|  | *SD* | 0.263 | 0.096 | 0.036 | 0.093 | 0.139 | 0.148 |
|  |  |  |  |  |  |  |  |
| B | *M* | -0.051 | 0.067 | 0.032 | 0.101 | 0.163 | 0.344 |
|  | *SD* | 0.191 | 0.197 | 0.051 | 0.241 | 0.102 | 0.258 |
|  |  |  |  |  |  |  |  |
| C | *M* | -0.478 | 0.494 | -0.009 | -0.135 | 0.283 | -0.122 |
|  | *SD* | 0.222 | 0.375 | 0.082 | 0.131 | 0.095 | 0.230 |
|  |  |  |  |  |  |  |  |
|  |  |  |  |  |  |  |  |
|  | *F(2, 207)* | 267.62 | 111.96 | 14.17 | 41.48 | 91.73 | 80.39 |
|  | *p* | **< .001** | **< .001** | **< .001** | **< .001** | **< .001** | **< .001** |
|  |  |  |  |  |  |  |  |
|  |  |  |  |  |  |  |  |
| B vs A | *M*_B_ - *M*_A_ | -0.45 | 0.16 | -0.01 | 0.21 | 0.14 | 0.33 |
|  |  | [-0.54, -0.35] | [0.07, 0.26] | [-0.03, 0.02] | [0.14, 0.27] | [0.09, 0.18] | [0.24, 0.41] |
|  | *p* _(Tukey adj.)_ | **< .001** | **< .001** | 0.70 | **< .001** | **< .001** | **< .001** |
|  |  |  |  |  |  |  |  |
|  |  |  |  |  |  |  |  |
| C vs A | *M*_C_ - *M*_A_ | -0.87 | 0.59 | -0.05 | -0.03 | 0.26 | -0.14 |
|  |  | [-0.96, -0.78] | [0.50, 0.68] | [-0.07, -0.03] | [-0.09, 0.03] | [0.21, 0.30] | [-0.22, -0.06] |
|  | *p* _(Tukey adj.)_ | **< .001** | **< .001** | **< .001** | 0.51 | **< .001** | **< .001** |
|  |  |  |  |  |  |  |  |
|  |  |  |  |  |  |  |  |
| C vs B | *M*_C_ - *M*_B_ | -0.43 | 0.43 | -0.04 | -0.24 | 0.12 | -0.47 |
|  |  | [-0.52, -0.33] | [0.32, 0.53] | [-0.07, -0.02] | [-0.30, -0.17] | [0.07, 0.17] | [-0.55, -0.38] |
|  | *p* _(Tukey adj.)_ | **< .001** | **< .001** | **< .001** | **< .001** | **< .001** | **< .001** |

Supplementary Table 3 T-test (Welch test) comparisons between subclusters: A1 vs A2, B1 vs B2 and C1 vs C2.

|  |  | **Wrestling vs. inactivity** | **Vocalising** | **Chasing** | **Non-interacting** | **Recurring Interactivity** | **Prolonged Interactivity** |
| --- | --- | --- | --- | --- | --- | --- | --- |
|  |  |  |  |  |  |  |  |
| A1 | *M* | 0.26 | -0.05 | 0.04 | -0.09 | 0.10 | 0.07 |
|  | *SD* | 0.19 | 0.08 | 0.04 | 0.09 | 0.10 | 0.12 |
| A2 | *M* | 0.70 | -0.19 | 0.04 | -0.15 | -0.13 | -0.11 |
|  | *SD* | 0.10 | 0.04 | 0.03 | 0.08 | 0.08 | 0.11 |
|  |  |  |  |  |  |  |  |
|  | *M*_2_ - *M*_1_ | 0.44 | -0.14 | 0.00 | -0.07 | -0.22 | -0.18 |
|  |  | [-0.50, -0.38] | [0.12, 0.17] | [-0.01, 0.02] | [0.03, 0.11] | [0.19, 0.26] | [0.13, 0.24] |
|  |  |  |  |  |  |  |  |
|  | *t* | -14.06 | 11.04 | 0.49 | 3.36 | 11.41 | 6.60 |
|  | df | 79.90 | 80.99 | 62.59 | 52.94 | 62.79 | 52.24 |
|  | *p* | **<.001** | **<.001** | .627 | **.001** | **<.001** | **<.001** |
|  |  |  |  |  |  |  |  |
|  |  |  |  |  |  |  |  |
| B1 | *M* | -0.10 | -0.01 | 0.02 | 0.34 | 0.15 | 0.13 |
|  | *SD* | 0.21 | 0.09 | 0.06 | 0.13 | 0.12 | 0.15 |
| B2 | *M* | -0.01 | 0.13 | 0.04 | -0.09 | 0.17 | 0.51 |
|  | *SD* | 0.17 | 0.23 | 0.04 | 0.08 | 0.08 | 0.19 |
|  |  |  |  |  |  |  |  |
|  | *M*_2_ - *M*_1_ | 0.09 | 0.14 | 0.03 | -0.43 | 0.02 | 0.38 |
|  |  | [-.019, 0.01] | [-0.23, -0.05] | [-0.06, 0.00] | [0.37, 0.49] | [-0.08, 0.04] | [-0.47, -0.30] |
|  |  |  |  |  |  |  |  |
|  | *t* | -1.78 | -3.17 | -2.15 | 14.72 | -0.66 | -8.66 |
|  | df | 47.62 | 43.43 | 43.97 | 37.12 | 41.61 | 56.84 |
|  | *p* | .081 | **.003** | **.037** | **<.001** | .510 | **<.001** |
|  |  |  |  |  |  |  |  |
|  |  |  |  |  |  |  |  |
| C1 | *M* | -0.40 | 0.29 | 0.00 | -0.09 | 0.28 | -0.11 |
|  | *SD* | 0.19 | 0.15 | 0.09 | 0.12 | 0.09 | 0.22 |
| C2 | *M* | -0.67 | 1.03 | -0.04 | -0.25 | 0.28 | -0.15 |
|  | *SD* | 0.17 | 0.22 | 0.06 | 0.04 | 0.12 | 0.25 |
|  |  |  |  |  |  |  |  |
|  | *M*_2_ - *M*_1_ | -0.27 | 0.74 | -0.05 | -0.17 | 0.00 | -0.04 |
|  |  | [0.17, 0.37] | [-0.85, -0.63] | [0.01, 0.08] | [0.13, 0.21] | [-0.06, 0.06] | [-0.10, 0.17] |
|  |  |  |  |  |  |  |  |
|  | *t* | 5.67 | -13.61 | 2.40 | 8.22 | -0.06 | 0.57 |
|  | df | 36.23 | 25.16 | 44.62 | 65.80 | 26.27 | 29.45 |
|  | *p* | **<.001** | **<.001** | **.021** | **<.001** | .954 | .572 |

Supplementary Table 4 Pearson chi-square tests for categorical variables of lower level hierarchical analysis´s clusters – L3, 2 d.f., * p≤0.05, ** p≤0.01, *** p≤0.001

| **L3** |  |  | **A1** | **A2** | **B1** | **B2** | **C1** | **C2** | **Results (χ^2^,p value)** |
| --- | --- | --- | --- | --- | --- | --- | --- | --- | --- |
| **Kittens** | Kittens | Observed (% of group) | 15 (26.3%) | 10 (38.5%) | 9 (34.6%) | 2 (6.1%) | 2 (4.1%) | 0 | χ2=28.578 |
|  |  | Expected | 10.3 | 4.7 | 4.7 | 6.0 | 8.9 | 3.4 |  |
|  |  | Standardised residual | 1.89 | **2.88** | **2.34** | -1.96 | **-2.91** | **-2.15** |  |
|  |  | p value on Standardised residual | 0.0589 | **0.0040**** | **0.0194*** | 0.0505 | **0.0036**** | **0.0317*** |  |
|  | Adults | Observed | 42 (73.7%) | 16 (61.5%) | 17 (65.4%) | 31 (93.9%) | 47 (95.9%) | 19 (100%) | p≤0.001 |
|  |  | Expected | 46.7 | 21.3 | 21,3 | 27,0 | 40,1 | 15,6 |  |
|  |  | Standardised residual | -1.89 | **-2.88** | **-2.34** | 1.96 | **2.91** | **2.15** |  |
|  |  | p value on Standardised residual | 0.0589 | **0.0040**** | **0.0194*** | 0.0505 | **0.0036**** | **0.0317*** |  |
| **Cats in original dyads** | In dyad | Observed | 44 (77.2%) | 22 (84.6%) | 10 (38.5%) | 14 (42.4%) | 24 (49.0%) | 8 (42.1%) | χ2=27.165 |
|  |  | Expected | 33.1 | 15.1 | 15.1 | 19.2 | 28.5 | 11.0 |  |
|  |  | Standardised residual | **3.42** | **2.93** | **-2.17** | **-1.99** | -1.48 | -1.48 |  |
|  |  | p value on Standardised residual | **0.0006***** | **0.0034**** | **0.0302**** | **0.0469*** | 0.1397 | 0.1386 |  |
|  | Not in dyad | Observed | 13 (22.8%) | 4  (15.4%) | 16 (61.5%) | 19 (57.6%) | 25 (51.0%) | 11 (57.9%) | p≤0.001 |
|  |  | Expected | 23.9 | 10.9 | 10.9 | 13.8 | 20.5 | 8.0 |  |
|  |  | Standardised residual | -3.42 | -2.93 | 2.17 | 1.99 | 1.48 | 1.48 |  |
|  |  | p value on Standardised residual | **0.0006***** | **0.0034**** | **0.0302**** | **0.0469*** | 0.1397 | 0.1386 |  |
| **Playful vs. Intermediate vs. Agonistic interaction** | Playful | Observed | 49 (86.0%) | 20 (76.9%) | 20 (76.9%) | 14 (42.4%) | 15 (30.6%) | 0 | χ2=101.02 |
|  |  | Expected | 32.0 | 14.6 | 14.6 | 18.5 | 27.5 | 10.7 |  |
|  |  | Standardised residual | **5.31** | **2.28** | **2.28** | -1.74 | **-4.12** | **-5.18** |  |
|  |  | p value on Standardised residual | **1.108468e-07***** | **0.0228*** | **0.0228*** | 0.0825 | **3.765014e-05***** | **2.263668e-07***** |  |
|  | Intermediate | Observed | 5 (8.8%) | 2 (7.7%) | 5 (19.2%) | 10 (30.3%) | 10 (20.4%) | 0 |  |
|  |  | Expected | 8.7 | 4.0 | 4.0 | 5.0 | 7.5 | 2.9 |  |
|  |  | Standardised residual | -1.59 | -1.14 | 0.61 | **2.62** | 1.15 | -1.94 | p≤0.001 |
|  |  | p value on Standardised residual | 0.1112 | 0.2537 | 0.5451 | **0.0087**** | 0.2501 | 0.0526 |  |
|  | Agonistic | Observed | 3 (5.3%) | 4 (15.4%) | 1 (3.8%) | 9 (27.3%) | 24 (49.0%) | 19 (100%) |  |
|  |  | Expected | 16.3 | 7.4 | 7.4 | 9.4 | 14.0 | 5.4 |  |
|  |  | Standardised residual | **-4.56** | -1.59 | **-2.98** | -0.18 | **3.61** | **7.23** |  |
|  |  | p value on Standardised residual | **5.027911e-06***** | 0.1118 | **0.0029**** | 0.8572 | **0.0003***** | **4.949374e-13***** |  |
| Total (N) | | | 57 | 26 | 26 | 33 | 49 | 19 |  |
